# Experimental Study of Children

**Published:** 1900-03

**Authors:** 


**Experimental Study of Children.** By ARTHUR MACDONALD. Washington: Government Printing Office. 1899.

It is desirable that we should have pointed out to us the necessity of complete methods for child study, both as an important branch of investigation into civilised man and for guidance in estimating the measure of the child's mental capabilities, and the proper relation to be observed between physical and mental culture. In three chapters from the Report of the Commissioner of the United States Bureau of Education, extending to 400 pages, Mr. MacDonald well says: "The establishment of the measure of work according to the strength of the individual is fundamental to the economy of health." He discusses methods of study, and lays much stress upon the value of anthropometry as affording a test for systems of physical culture. The author personally studied 1,074 children in the Washington schools, considering cephalic index, and sensibility to heat and locality in relation to mental ability, sociological condition, sex and puberty; whilst anthropometrical, sociological, and psychological study of nearly 22,000 children was made by their teachers under careful supervision. Lastly, an investigation into abnormal children was carried out. The following are some of the main conclusions arrived at by the author: Dolichocephaly increases as mental ability decreases; dull and unruly boys seem to have a large percentage of long heads; the children of non-labouring classes are more sensitive to heat and locality on skin than those of labouring classes. From study of the whole mass of children it appears that as the circumference of head enlarges mental ability increases; that girls are heavier and taller for a certain time, one or two years, before puberty; that bright boys are in general taller and heavier than dull boys; that a mixture of nationalities seems unfavourable to the development of mental ability. As regards the abnormal children, boys of non-labouring classes show a higher percentage of sickliness than those of the working orders; defects of speech are more common in boys than girls; there is more unruliness in boys, especially the dull ones; abnormalities are most frequent at dentition and puberty; such children are inferior in height, sitting-height, and head-circumference to children in general. A great number of investigations made by acknowledged experts supplement the author's own studies. Bearing especially on his remarks concerning a necessary parallelism to be kept between mental and physical culture are Dr. Porter's conclusions, derived from examination into 33,500 children. These are, in general terms, that mediocrity of mind is associated with mediocrity of physique, and that no child whose weight is below the average for its age should, as a rule, be put into a school-grade beyond the average of its age, except after physical examination.

To all those engaged in psychological study, with either special or general aims, this book should appeal. The author has been markedly successful in giving everywhere a clear analysis of the enormous amount of statistical matter.

